# Prognostic factors in Sézary syndrome - a retrospective propensity score-matched study on 1277 patients

**DOI:** 10.3389/fimmu.2026.1747618

**Published:** 2026-03-19

**Authors:** Nadia Ninosu, Max Kappenstein, Niklas Gebauer, Dagmar von Bubnoff, Nikolas von Bubnoff, Jan P. Nicolay

**Affiliations:** 1 Department of Dermatology, Allergology and Venerology, University Medical Center Mannheim/Ruprecht Karls University of Heidelberg, Mannheim, Germany; 2 Department of Nuclear Medicine, University Hospital Frankfurt am Main, Frankfurt am Main, Germany; 3 Department of Hematology and Oncology, University Hospital of Schleswig-Holstein, Lübeck, Germany; 4 Department of Dermatology, Allergology and Venerology, University Hospital Schleswig-Holstein, Lübeck, Germany; 5 Skin Cancer Unit, German Cancer Research Center, Heidelberg, Germany

**Keywords:** CTCL, cutaneous T-cell lymphoma, prognostic factors, risk factors, Sézary syndrome, anemia, TriNetX, real-world data

## Abstract

**Introduction:**

Sézary syndrome (SS) is a rare neoplasm associated with poor prognosis. Due to the rarity of the disease, large-scale investigations are scarce, while identifying prognostic factors is essential for improving treatment outcomes. The primary objective was to investigate prognostic factors in a large population of SS patients on survival and infections. The secondary objective was to validate a previously suggested prognostic index model.

**Methods:**

We conducted a propensity score-matched retrospective cohort study using real-world data from 1277 patients in the TriNetX database. Risk factors examined included age, elevated lactate dehydrogenase (LDH), anemia, elevated white blood cell count (WBC), sex, and race. Examined outcomes were: 5-year survival, Systemic Inflammatory Response Syndrome (SIRS), sepsis and pneumonia. The prognostic index model was simplified into factors assessable through TriNetX.

**Results:**

Age > 60 years, elevated LDH, anemia and elevated WBC were risk factors for 5-year overall survival. Elevated LDH, anemia, elevated WBC and African American/black race were significantly associated with sepsis. Anemia and elevated WBC were also linked to higher risk of pneumonia. Additionally, we devised and validated a simplified prognostic index model (sPIM) accounting for patient focus on SS (stage IV) patients.

**Conclusion:**

In the largest group of SS patients assessed for risk stratification to-date, we confirm age and elevated LDH as risk factors in SS and their applicability in the prognostic index model. This study introduces anemia as a novel risk factor and demonstrates the prognostic value of leukocytosis in SS. While race requires further investigation, sex appears to have no significant impact on survival.

## 1 Introduction

Sézary syndrome (SS) is an aggressive and leukemic subtype of primary cutaneous T-cell lymphoma (CTCL), a group of rare non-Hodgkin lymphomas that mainly affect the skin (1–4). SS is characterized by its systemic involvement and unfavorable clinical prognosis. It manifests with erythroderma, lymphadenopathy and the presence of neoplastic T-cells - known as Sézary cells - in the peripheral blood (5). Clinical progression leads to a higher susceptibility for infections, which represent a primary cause of mortality in patients with SS (6, 7).

Although SS accounts for only about 5% of all CTCL cases, it is associated with the poorest outcome, including a median survival of less than five years (8). Due to the poor prognosis, identifying risk factors that contribute to disease progression and mortality is critical for improving patient management and tailoring therapeutic strategies. Current risk stratification for mycosis fungoides (MF), the most common non-leukemic CTCL, and SS are primarily based on the TNMB staging system. Hereby, SS is per se classified as stage IV disease (9).

To improve prognostic assessment, Benton et al. developed the prognostic index CLIPi (10), which used the CLIC study cohort with the most comprehensive dataset of patients with Stage IV CTCL. The study included 498 stage IV patients and, in addition to advanced stage, identified age over 60 years, large-cell transformation (LCT), and elevated lactate dehydrogenase (LDH) levels as independent prognostic factors (11). Those were combined in a prognostic index model as a simple and reproducible survival benchmark where zero or one variable equals low risk, two variables equal intermediate risk, and three to four variables equal high risk (11). Recently, this index was revised, and instead of the advanced disease stage, the N3 nodal status was added (12). This recent adjustment further highlights that the TNMB staging system is inadequate for risk stratification, emphasizing the importance of investigating additional risk factors.

Other potential prognostic markers were reported, including male sex, histologic features of folliculotropism (FT), CD30 positivity, high white blood cell count (WBC), and elevated β2-microglobulin level (13–19). Moreover, African American (AA)/black patients with MF/SS had adverse prognosis compared to Caucasian/white patients (20–23). Since CTCL is associated with impaired hematopoiesis (24), signs of bone marrow insufficiency, such as anemia, could be further poor prognostic factors. However, to our knowledge, anemia has previously not been described as prognostic factor in SS.

Due to the rarity of the disease, large-scale studies are limited, making the identification of prognostic factors challenging. However, these factors are crucial for improving therapeutic management and treatment outcomes. With the development of extensive real-world databases, such as TriNetX, it has become possible to analyze larger patient cohorts and investigate potential risk factors with greater statistical power. By utilizing the TriNetX platform, we aimed to validate established risk factors and identify new potential prognostic markers.

## 2 Methods

### 2.1 Data source

The TriNetX federate research network was used for this study, which holds a collection of electronic medical records (EMRs) from 163 healthcare organizations (HCOs) worldwide at the time of data analysis. The network contains detailed EMR data on diagnoses, procedures, medications, laboratory results and partial genomic information for approximately 190 million patients of whom the majority (≈130 million) are derived from the U.S. collaborative network.

### 2.2 Structure of the study

This is a retrospective analysis in which patients were identified at the time of their initial diagnosis of SS (ICD-10 code C84.1) and their outcomes were compared using TriNetX EMR data with cohorts matched through propensity score matching (PSM) as reported previously (25) in order to assess the impact of each factor independently of other variables and to minimize confounding. Patients of at least 18 years diagnosed with SS were included. Diagnosis of SS was defined as index event. Only patients with SS diagnosis established for more than 5 years and with at least one documented visit after the 5 year observation period or with recorded death within the TriNetX EMR data were included in order to ensure adequate documentation of the disease course during the observation period, avoiding disproportional inclusion of individuals with poor outcome due to over-proportional censoring of patients with survival longer than recorded. A sensitivity analysis without this requirement is presented in **Supplementary Table 1**. TriNetX excludes patients that met the index event (SS diagnosis) more than 20 years before registration. PSM was performed for sex, race, LDH, hemoglobin and WBC to reduce potential confounding. Separate PSM cohorts were generated for each risk factor. The platform uses 1:1 nearest neighbor matching with a caliper of 0.1. Standardized differences before and after matching are included in **Supplementary Table 2**.

The potential risk factors assessed were age, anemia, LDH, WBC, race and sex. Anemia was defined by the updated classification of WHO 2024 and therefore assessed separately for men and women due to diverging hemoglobin thresholds (26). 250 U/l was chosen as threshold for elevated LDH following Xu et al. (19). Due to a limited number of entries within TriNetX, it was not possible to evaluate the additional risk factors such as large cell transformation or N3 nodal status mentioned in the introduction in a robust manner. Baseline laboratory values for cohort definitions were obtained one week before or after the index event. For PSM, the most recent value within a week before the index event was selected; PSM included matching by the number of patients with available laboratory values.

For each parameter assessed, the patients were classified in two cohorts by criteria indicated in **Table 1**.

**Table 1:** Definition of assessed cohorts.

Risk factor	Cohort 1	Cohort 2
Stage	IV	IV
Age	≤60 years	>60 years
LDH	≤250	>250
Anemia (Hb)	≥12 g/dL (female) ≥13 g/dL (male)	<12 g/dL (female) <13 g/dL (male)
Leukocytes	≤11 × 10^3^/µL	>11 × 10^3^/µL
Race	White/Caucasian	Black/AA
Sex	Women	Men

The assessed outcomes were death, and, due to the high impact of infections on SS patient survival (7, 27), SIRS (Systemic Inflammatory Response Syndrome) (ICD-10 code R65), sepsis (ICD-10 code A41) and pneumonia (ICD-10 code J18). Outcomes were assessed independently for each risk factor.

The data accessed through TriNetX are provided in an aggregated format and consist solely of anonymized information that meets the de-identification standards of the US Health Insurance Portability and Accountability Act (HIPAA) section §164.514(a). Because only de-identified and anonymized EMR were used in this study, Institutional Review Board or ethics approval and written informed consent were not required.

### 2.3 Statistical analysis

Statistical analyses were performed using the TriNetX analytics platform. Differences in survival were assessed using Kaplan-Meier analysis, with the log-rank test used to assess statistical significance between outcomes considering the timing of events. Risk ratios (RR) and corresponding 95% confidence intervals (CI 95%) for the respective outcomes were calculated for each cohort, with RRs representing the relative risk for the respective event in cohort 1 compared to cohort 2 without consideration of timeliness. A Cox-regression proportional hazard model was used to calculate hazard ratios with 95% CIs for the incremental risk of death from age 60 onwards. A p-value of ≤ 0.05 was considered statistically significant.

To evaluate the validity of the prognostic index model established by Scarisbrick et al. (11), we established a simplified prognostic index model (sPIM) for criteria available through TriNetX, thus excluding LCT. One point was the lowest possible score in the present study as the patient population contained only SS patients, by definition fulfilling the Stage IV criterion. For every other fulfilled criterion (age ≥ 60 years, elevated LDH **>** 250), one point was assigned, making 3 points the highest possible score. No PSM was performed for the simplified score comparison to assess its validity in clinical practice without considering further parameters. An overview of the 2015 Prognostic Index Model, the revised 2025 New Prognostic Index (CLIPI) and the sPIM is shown in **Table 2**.

**Table 2:** Comparison of prognostic models in advanced stage CTCL.

Criterion	2015 prognostic index model (11)	2025 new prognostic index (CLIPI) (12)	Simplified prognostic index model (sPIM)
Patient Population	Advanced-stage (IIB–IVB)	Advanced-stage (IIB–IVB)	Stage IV, corresponding to Sézary Syndrome (SS) patients
Prognostic Factors	1. Stage IV 2. Age > 60 years 3. Elevated serum LDH 4. Large cell transformation	1. N3 node status 2. Age > 60 years 3. Elevated serum LDH 4. Large cell transformation	1. Stage IV (SS) 2. Age > 60 years 3. Elevated LDH (> 250)
Risk Grouping	Low: 0–1 factor Intermediate: 2 factors High: 3–4 factors	Low: 0–1 factor Intermediate: 2 factors High: 3–4 factors	Score 1: Stage IV only Score 2: + 1 factor Score 3: All factors

## 3 Results

At the time of data analysis, the network included a total of 3,111 patients diagnosed with SS. 1277 patients from 76 HCOs met the inclusion criteria. Patient characteristics of this cohort are shown in **Table 3**. Shown p-values refer to log-rank test.

**Table 3:** Characteristics of the study cohort.

Attribute	Value
Total cohort, n (%)	1,277 (100%)
Sex, n (%)	
Male	612 (47.9%)
Female	499 (39.1%)
Unknown	166 (13.0%)
Age at index (mean; in years ± SD)	59.2 ± 13.9
Race, n (%)	
White/Caucasian	725 (56.77%)
Unknown and other	321 (25.14%)
African American/Black	210 (16.44%)
Asian	21 (1.64%)
Laboratory (mean ± SD)	
LDH (enzymatic activity/volume) in blood	342.3 ± 461.6
Hemoglobin (in g/dL) in blood (women)	12.0 ± 1.9
Hemoglobin (in g/dL) in blood (men)	12.9 ± 2.3
Leukocytes (in ×10³/μL) in blood	26.8 ± 273.9
Outcome	
Five-year survival rate (in %)	57.04%
Documented five-year SIRS rate (in %)	13.07%
Documented five-year sepsis rate (in %)	22.58%
Documented five-year pneumonia rate (in %)	19.00%

### 3.1 Independent impact of parameters

#### 3.1.1 Impact of age

**Figure 1:**
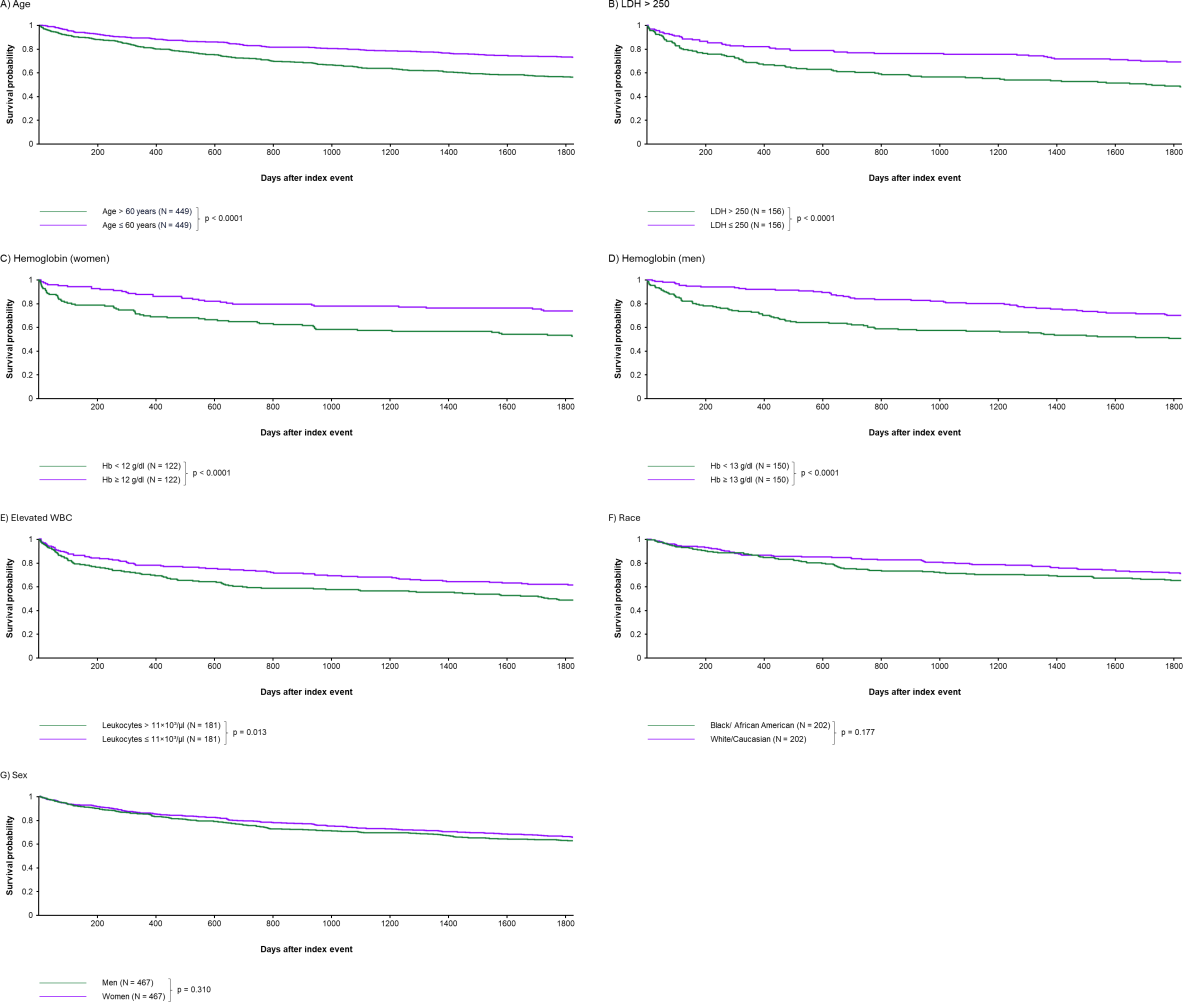
Independent impact of age and laboratory parameters on 5-year survival of patients with Sézary Syndrome (SS). Age > 60 years **(A)**, lactate dehydrogenase (LDH) >250 U/l **(B)**, anemia with Hb < 12 g/dL (women) **(C)**, anemia with Hb < 13 g/dL (men) **(D)**, elevated white blood cell count (WBC) > 11 × 10^3^/µL **(E)**, are independent criteria for survival [with *p* < 0.0001 for **(A-D)** and *p* = 0.013 for **(E)**]. There was no difference in 5 year-survival between black/African American and white/Caucasian patients (*p* = 0.177) **(F)** or men and women (*p* = 0.310) **(G)**.

PSM divided the study population into two cohorts of 449 patients each. Age > 60 years was associated with significantly lower survival (RR 0.617, CI 95% 0.513-0.743, **Table 4**; survival 72.96% vs. 56.15% % at five years, p < 0.0001, **Figure 1A**). Age was also found to be a significant factor as a continuous variable (Hazard Ratio 1.032; 95% CI 1.019-1.046; p < 0.0001), indicating that, from age 60, the hazard increased by 3.2% for each additional year of age. There was no significant correlation between age and the risk for SIRS (RR 1.294, CI 95% 0.920-1.820; p = 0.283), sepsis (RR 1.114, CI 0.862-1.439; p = 0.691) and pneumonia (RR 1.052; 95% CI 0.792-1.396; p = 0.898).

**Table 4:** Risk Ratio (RR), 95% Confidence Interval (95% CI) and p-value (p) from the log-rank test for impact of independent parameters (rows) on events (columns) double for patients with SS within 5 years post-diagnosis.

Risk factor	Death			SIRS		Sepsis				Pneumonia		
	RR	95% CI	p	RR	95% CI	p	RR	95% CI	p	RR	95% CI	p
Age	0.617	0.513-0.743	< 0.0001	1.294	0.920-1.820	0.283	1.114	0.862-1.439	0.691	1.052	0.792-1.396	0.898
LDH	0.593	0.448-0.784	< 0.0001	0.595	0.368-0.960	0.009	0.623	0.428-0.905	0.002	0.974	0.656-1.445	0.435
Anemia (female)	0.552	0.388- 0.784	< 0.0001	0.375	0.203-0.693	< 0.0001	0.475	0.292-0.772	< 0.0001	0.567	0.330-0.972	0.006
Anemia (male)	0.608	0.453-0.815	< 0.0001	0.826	0.470-1.452	0.218	0.674	0.454-1.001	0.006	0.618	0.377-1.013	0.011
WBC	0.753	0.597-0.949	0.0013	0.750	0.476-1.182	0.125	0.621	0.446-0.865	0.001	1.051	0.714-1.548	0.824
Race	0.829	0.621-1.105	0.177	0.500	0.307-0.813	0.003	0.547	0.380-0.786	<0.0001	0.700	0.450-1.089	0.071
Sex	0.926	0.780-1.098	0.310	1.314	0.934-1.847	0.200	1.156	0.899-1.486	0.423	1.217	0.910-1.629	0.275

#### 3.1.2 Impact of LDH

Here, PSM resulted in two groups of 156 patients each. LDH greater than 250 was associated with significantly lower survival (RR 0.593, CI 95% 0.448-0.784, **Table 4**; survival 69.07% vs 48.06% at five years, p < 0.0001, **Figure 1B**) as well as significantly higher risk for SIRS (RR 0.595, CI 95% 0.368-0.960; p = 0.009) and sepsis (RR 0.623, CI 95% 0.428-0.905; p = 0.002). Pneumonia was not significantly affected (RR 0.974, CI 95% 0.656-1.445; p = 0.435).

#### 3.1.3 Impact of anemia

##### 3.1.3.1 Women

A total of 122 women in each group were included following PSM. Anemia (Hb < 12 g/dl) was associated with significantly higher risk of death (RR 0.552, CI 95% 0.388- 0.784; **Table 4**, survival 73.77% vs 52.45% at five years, p < 0.0001, **Figure 1C**), as well as significantly higher risk of SIRS (RR 0.375, CI 95% 0.203-0.693; p < 0.0001), sepsis (RR 0.475, CI 95% 0.292-0.772; p < 0.0001) and pneumonia (RR 0.567, CI 95% 0.330-0.972; p = 0.006).

##### 3.1.3.2 Men

A total of 150 men in each group were included following PSM. Anemia (Hb < 13 g/dl) was associated with significantly higher risk of death (RR 0.608, CI 95% 0.453-0.815; **Table 4**, survival 70.00% vs 50.56% at five years, p <0.0001, **Figure 1D**), as well as significantly higher risk of sepsis (RR 0.674, CI 95% 0.454-1.001; p = 0.006). The risk for pneumonia is significantly higher (p = 0.011), yet with RR of 0.618 with CI 95% (0.377-1.013). The risk of SIRS was not significantly higher (RR 0.826, CI 95% 0.470-1.452; p = 0.218).

To account for the possible induction of anemia by previous or concurrent therapies and the possible association with an advanced disease stage, our analysis was based on laboratory values recorded within one week before and after the first documented diagnosis of SS. Additionally, in a secondary analysis, we excluded common systemic therapies for MF and Sézary syndrome as prior treatments. Anemia remained a significant adverse prognostic factor also after excluding systemic therapies. The following treatments were excluded in the secondary analysis: Extracorporeal photopheresis, methotrexate, bexarotene, vorinostat, mogamulizumab, gemcitabine, brentuximab vedotin, acitretin, pralatrexate, alemtuzumab, chlorambucil, doxorubicin, romidepsin, etoposide, interferons, bone marrow transplant status and radiotherapy. These findings are detailed in the **Supplementary Materials** (**Supplementary Figure 1**).

#### 3.1.4 Impact of WBC

A total of 181 patients were matched and analyzed per group. Leukocytosis (WBC > 11×10³/µl) was associated with lower survival (RR 0.753, CI 95% 0.597-0.949, **Table 4**; survival 61.32% vs. 48.62% at five years, p = 0.0013, **Figure 1E**) and sepsis (RR 0.621, CI 95% 0.446-0.865; p = 0.001), but not with a higher risk of SIRS (RR 0.750, CI 95% 0.476-1.182; p = 0.125), or pneumonia (p = 0.824; RR 1.051, CI 95% 0.714-1.548).

#### 3.1.5 Impact of race

Since 321 (25.14%) patients were of unknown and other race and only 21 (1.64%) were Asian, the analysis was restricted to a comparison between white/Caucasian and AA/black patients. Following PSM, a total of 202 patients were included. Black/AA race was not associated with a significantly higher risk of death (RR 0.829, CI 95% 0.621-1.105), **Table 4**, survival 71.21% vs 65.35% at five years, p=0.177, **Figure 1F**) but with a significantly higher risk of SIRS (RR 0.500, CI 95% 0.307-0.813; p = 0.003) and sepsis (RR 0.547, CI 95% 0.380-0.786; p < 0.0001). No risk difference was observed for pneumonia (RR 0.700, CI 95% 0.450-1.089; p = 0.071).

#### 3.1.6 Impact of sex

After PSM, 467 patients were included in each group. Survival outcomes did not differ significantly between men and women (RR 0.926, CI 95% 0.780-1.098, with survival rates at five years of 65.27% for women compared to 62.41% for men, p = 0.310 **Figure 1G**). Similarly, sex did not show any significant association with SIRS (RR 1.314, CI 95% 0.934-1.847; p = 0.200), sepsis (RR 1.156, CI 95% 0.899-1.486; p = 0.423) and pneumonia (RR 1.217, CI 95% 0.910-1.629; p = 0.275).

### 3.2 Impact of the sPIM

#### 3.2.1 Comparison of 1 vs. 2 points

**Figure 2:**
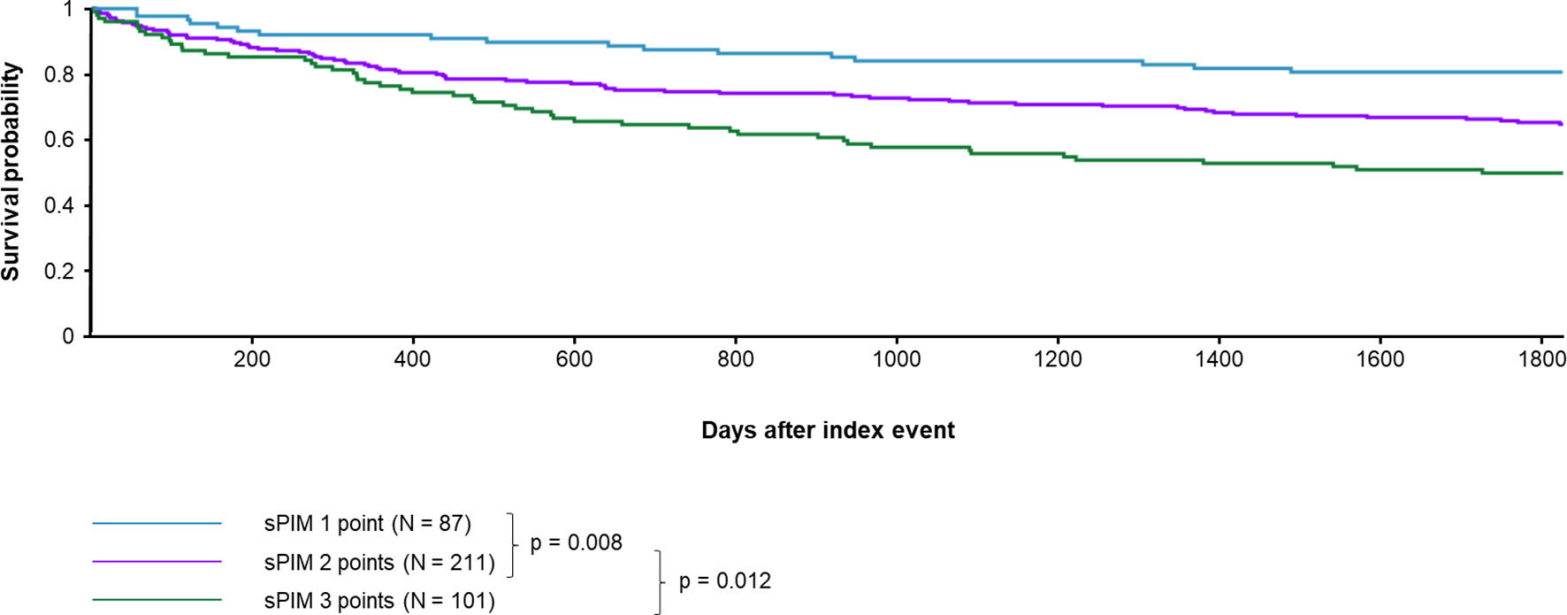
Difference in 5-year survival for patients with simplified prognostic index model (sPIM) scores from 1 to 3. Patients with 1 sPIM point showed longer survival than patients with 2 sPIM points (*p* = 0.008). Patients with 2 sPIM points showed longer survival than patients with 3 sPIM points (*p* < 0.012).

Patients with 2 points (vs. 1 point) showed a higher risk of death (RR 0.565 CI 95% 0.355- 0.899, **Table 5**; survival 80.46% vs. 64.51% at five years, p = 0.008, **Figure 2**).

**Table 5:** Risk Ratio (RR), 95% Confidence Interval (95% CI) and p-value (p) from the log-rank test for impact of the sPIM score on events for SS patients within 5 years post-diagnosis.

Death			
Simplified prognostic index model	RR	(95% CI)	p
1 vs. 2	0.565	(0.355- 0.899)	0.008
2 vs. 3	0.685	(0.524-0.896)	0.0012

#### 3.2.2 Comparison of 2 vs. 3 points

Patients with 3 points (vs. 2 points) showed significantly higher risk of death (RR 0.685,

CI 95% 0.524-0.896, **Table 5**; survival 64.51% vs. 49.50% at five years, p = 0.0012, **Figure 2**).

## 4 Discussion

To our knowledge, this study represents the largest dataset investigating risk factors in patients with SS to date (N = 1277). The presented data confirm the impact of the established risk factors age, elevated LDH, and elevated WBC (11, 16, 19). In addition, we identified anemia as a novel potential risk factor.

Our analysis confirmed higher age as risk factor for survival. In previous studies, age has consistently been linked to lower survival in MF/SS (11, 14, 16, 28). Age had a significant impact on the 5-year survival. Moreover, the mortality increased by 3.2% per additional year from the age above 60. These findings align closely with the CLIC study which reported a 3% annual increase in risk for mortality in a similar model, but in a different SS patient population (11). In our dataset, the link of age with a higher risk of SIRS, sepsis or pneumonia was not significant, which indicates a direct adverse effect of age on survival in SS, but also could be attributed to a higher risk of elderly patients to die from other comorbidities, whereas infections could be observed at similar rates among younger patients with SS.

An elevated LDH has previously been identified as a prognostic factor in CTCL (11, 16, 19). In the present study, LDH > 250 had a significant impact on 5-year-survival as well as SIRS and sepsis risk. Elevated LDH levels are common in many malignancies and indicate cell turnover, contributing to the acidification of the tumor microenvironment, promoting angiogenesis, tumor growth, and metastasis (29). High LDH levels are recognized as a poor prognostic indicator in non-Hodgkin lymphoma (30) and germ cell tumors (31) and are part of prognostic indices for aggressive follicular and mantle-cell lymphomas (32, 33) as well as non-seminoma (34).

We could identify anemia as an additional risk factor. The presence of anemia had a significant adverse impact on 5-year-survival, sepsis and pneumonia, and, in female patients, on SIRS. Anemia remained an adverse prognostic factor after the exclusion of patients who had previously received systemic therapies for SS. Patients with CTCL exhibit impaired hematopoiesis (24), suggesting that anemia can be interpreted as a sign of advanced disease. Additionally, anemia is frequently observed in critically ill patients and is associated with complications such as organ hypoxia, myocardial infarction, stroke and has been linked to respiratory dysfunction and increased mortality (35–37). Alternatively, anemia could serve as an indicator of other underlying factors, such as comorbidities or persistent chronic inflammation (38).

In the present study, anemia had a significant impact on the risk of severe infections. Infections represent a primary cause of mortality in patients with MF and SS (6, 7). Additionally, numerous previous studies linked anemia to an adverse outcome in patients with pneumonia (39, 40) and sepsis (41, 42). Blaizot et al. (2018) reported that skin infections were the most frequently observed type of infectious event (35%) and pneumonia the second most common cause (31%), while pneumonia is the main cause of sepsis in the general population (43, 44). Based on our findings, we recommend a multicentric, prospective study to confirm the presented observations on anemia and to specify further implications on the course of the disease.

Our analysis showed a significant impact of elevated WBC on overall survival and sepsis. No further analysis on Sézary cells were included in the present study as TriNetX, at the time of the publication of this study, does not provide sufficient data on Sézary cells; however, leukocytes could serve as surrogate for an increased tumor burden. The results of the present study are in line with the CLIC study (11). However, we cannot exclude that other white blood cells such as neutrophils may have contributed to increased WBC in this cohort.

MF and SS are more common among AA/black individuals compared to white patients in the US (3, 45, 46), with frequently observed worse outcomes (20–23) and increased inpatient healthcare utilization (47). Genetic, immunological, or molecular factors may contribute to these disparities (48). Unique proinflammatory pathways in black individuals may predispose inflammatory skin conditions, increasing sepsis risk in case of skin barrier disruption and inflammation in CTCL, which is underscored by both our study and Hooper et al.’s findings (49). While socioeconomic factors have been proposed as drivers of racial disparities in survival (21, 50), other studies present conflicting results, suggesting that rather clinicopathologic features than socioeconomic drivers contribute to survival and disease progression.

The small proportion of AA/black patients in our dataset suggests that such individuals may be underrepresented in large treatment centers and databases like TriNetX. Additionally, our study did not account for socioeconomic factors, which could further limit the interpretation of survival disparities.

No unambiguous results were obtained for the impact of sex. A limited number of previous studies showed a relevantly poorer survival in men (10, 16). Our analyses could not find a significant impact and are consistent with the CLIC data (11).

The identification of risk determinants allows for the stratification of patients for the adjustment of therapy and clinical monitoring. The publications by Benton et al. and Scarisbrick et al. represented milestones by identifying additional prognostic factors beyond the TNMB staging system, indicating the requirement of further validation of this index in larger cohorts (10, 11). Several subsequently conducted studies reported conflicting data. In Brazil, a study of 102 stage IA/IB MF patients found no significant progression or mortality differences between risk groups (51). Similarly, Sanz-Bueno et al. reported higher survival in the intermediate-risk group than the low-risk group, but overall survival was lowest in high-risk patients, though not significantly (52). Nikolaou et al. found high-risk patients had significantly higher progression and mortality, while intermediate-risk patients showed no significant differences from low-risk patients, highlighting a potential limitation in stratifying risk for intermediate cases (53). Danish et al. confirmed the CLIPi score’s validity in 390 patients (54). Our study, using a simplified model applied to SS patients, found that higher scores correlated with poorer survival, aligning with the CLIC data. However, a revised version of the CLIPi score was suggested recently, adding N3 lymph node state instead of advanced disease stage as prognostic factor in order to improve the prognostic accuracy of the index (12). As lymph node involvement is not sufficiently documented in TriNetX, further research in sufficiently large cohorts is necessary to assess how the prognostic factors in the revised CLIPi score including lymph node involvement as well as large-cell transformation and the present study possess combined prognostic value.

The present study has several limitations. The used EMR data could include misdiagnoses or miscoding and may not fully account for all confounders. Additionally, the observed 5-year survival is likely overestimated, as patients deceased outside of the TriNetX-linked institution might not have been consistently recorded in the database, leading to a bias towards patients with longer survival as outcomes of patients with follow-up after five years were likely more consistently reported. The present study indicates a 5-year survival rate of 57.04% for SS (stage IV) patients, while previous studies reported survival rates between 23.3%-52.5% depending on disease stage (55), which seems to confirm the indicated bias towards reporting of patients with longer survival. However, there is no indication for a systemic bias in capturing patient outcomes between cohorts.

Moreover, prior treatments may not have been consistently documented in TriNetX. However, the large dataset and the definition of the first SS diagnosis as index event provide a robust framework for the analysis. Importantly, our findings align closely with those from the CLIC (11) study which supports the reliability and quality of documentation within the TriNetX database for this patient cohort.

In conclusion, the findings of our study could help guide earlier and more aggressive treatment for SS patients with risk factors predicting adverse outcome, as well as the implementation of closer, more frequent monitoring to allow early detection of disease progression. Our analysis provides empirical evidence that age, elevated LDH, and leukocytosis are independent prognostic factors in SS patients. Additionally, our findings suggest that anemia should be considered as a potential adverse prognostic factor.

## Data Availability

The data analyzed in this study is subject to the following licenses/restrictions: The data was pulled from the constantly updated platform TriNetX and represents the status as of October 2025. Access: https://live.trinetx.com/. Requests to access these datasets should be directed to nadia.ninosu@umm.de.
