# Supplementary material for: Prognostic factors in Sézary syndrome - a retrospective propensity score-matched study on 1277 patients
Source: Front Immunol. 2026 Mar 19;17:1747618. doi: 10.3389/fimmu.2026.1747618 (PMC13043635; doi:10.3389/fimmu.2026.1747618)
Supplement: Supplementary file 1 [file DataSheet1.pdf]

Figure S1: Differences in survival in patients without previous systemic therapies *

A) Hemoglobin (women)

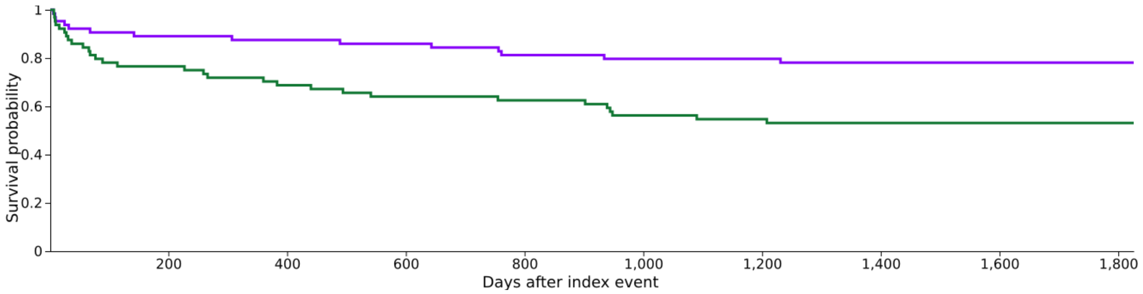

Hb < 12 g/dl (N = 64)
Hb ≥ 12 g/dl (N = 64)
p = 0.003

B) Hemoglobin (men)

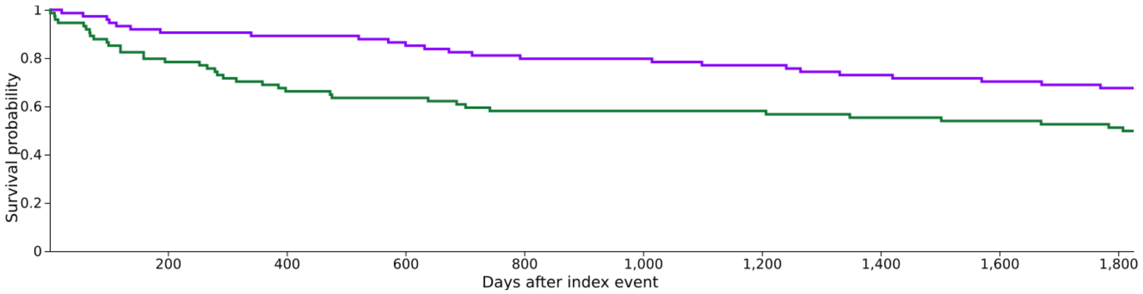

Hb < 13 g/dl (N = 74)
Hb ≥ 13 g/dl (N = 74)
p = 0.013

*The following treatments were excluded: Extracorporeal photopheresis, methotrexate, bexarotene, vorinostat, mogamulizumab, gemcitabine, brentuximab vedotin, acitretin, pralatrexate, alemtuzumab, chlorambucil, doxorubicin, romidepsin, etoposide, interferons, bone marrow transplant status and radiotherapy.
