# Supplementary material for: Prognostic factors in Sézary syndrome - a retrospective propensity score-matched study on 1277 patients
Source: Front Immunol. 2026 Mar 19;17:1747618. doi: 10.3389/fimmu.2026.1747618 (PMC13043635; doi:10.3389/fimmu.2026.1747618)
Supplement: Supplementary file 2 [file Table1.docx]

**Supplementary Table 1:** Sensitivity analysis. Risk Ratio (RR), 95% Confidence Interval (95% CI) and p-value (p) from the log-rank test for impact of independent parameters (rows) on events (columns) on events for patients with SS within 5 years post-diagnosis without required 5-year follow-up or documented death. The data were retrieved in February 2026.

|  | **Death** | | | **SIRS** | | |  | **Sepsis** | | | | **Pneumonia** | | | |
| --- | --- | --- | --- | --- | --- | --- | --- | --- | --- | --- | --- | --- | --- | --- | --- |
| **Risk Factor** | **RR** | **95% CI** | **p** | **RR** | **95% CI** | **p** | | | **RR** | **95% CI** | **p** | | **RR** | **95% CI** | **p** |
| **Age** | 0.545 | 0.427–0.697 | <0.001 | 1.302 | 0.893–1.899 | 0.340 | | | 0.987 | 0.738–1.320 | 0.580 | | 1.000 | 0.706–1.417 | 0.585 |
| **LDH** | 0.455 | 0.314–0.658 | <0.001 | 0.533 | 0.303–0.938 | 0.005 | | | 0.622 | 0.410–0.943 | 0.003 | | 0.875 | 0.555–1.380 | 0.140 |
| **Anemia (female)** | 0.593 | 0.413–0.851 | 0.001 | 0.382 | 0.212–0.690 | <0.001 | | | 0.500 | 0.310–0.806 | <0.001 | | 0.452 | 0.252–0.808 | 0.001 |
| **Anemia (male)** | 0.587 | 0.414–0.832 | <0.001 | 0.880 | 0.516–1.501 | 0.289 | | | 0.821 | 0.540–1.247 | 0.087 | | 0.846 | 0.499–1.435 | 0.197 |
| **WBC** | 0.744 | 0.568–0.975 | 0.003 | 0.884 | 0.593–1.316 | 0.233 | | | 0.710 | 0.516–0.978 | 0.005 | | 1.175 | 0.802–1.722 | 0.968 |
| **Race** | 0.980 | 0.693–1.385 | 0.833 | 0.667 | 0.412–1.078 | 0.088 | | | 0.574 | 0.385–0.856 | 0.004 | | 0.935 | 0.585–1.497 | 0.729 |
| **Sex** | 0.970 | 0.786–1.197 | 0.506 | 1.120 | 0.780–1.608 | 0.747 | | | 0.988 | 0.747–1.307 | 0.697 | | 1.188 | 0.825–1.709 | 0.497 |
