# Supplementary material for: Prognostic factors in Sézary syndrome - a retrospective propensity score-matched study on 1277 patients
Source: Front Immunol. 2026 Mar 19;17:1747618. doi: 10.3389/fimmu.2026.1747618 (PMC13043635; doi:10.3389/fimmu.2026.1747618)
Supplement: Supplementary file 3 [file Table2.docx]

**Supplementary Table 2:** Characteristics before and after propensitiy score matching for prognostic factor cohorts

**A) Age**

| **Cohort 1 (N = 648) and cohort 2 (N = 687) characteristics before propensity score matching** | | | | | | | | | |
| --- | --- | --- | --- | --- | --- | --- | --- | --- | --- |
| **Demographics** | | | | | | | | |  |
|  | Cohort | |  | Mean ± SD | Patients |  | P-Value | Std diff. |  |
|  | 1 2 |  | Age at Index | 47.5 +/- 10.9 68.8 +/- 5.9 | 517 649 |  | <0.001 | 2.427 |  |
|  | 1 2 |  | White |  | 288 471 |  | <0.001 | 0.357 |  |
|  | 1 2 |  | Unknown Race |  | 69 73 |  | 0.276 | 0.064 |  |
|  | 1 2 |  | Female |  | 219 297 |  | 0.245 | 0.069 |  |
|  | 1 2 |  | Black or African American |  | 142 81 |  | <0.001 | 0.382 |  |
|  | 1 2 |  | Male |  | 298 352 |  | 0.245 | 0.069 |  |
|  | 1 2 |  | Asian |  | 10 11 |  | 0.760 | 0.018 |  |
| **Laboratory** | | | | | | | | |  |
|  | Cohort | |  | Mean ± SD | Patients |  | P-Value | Std diff. |  |
|  | 1 2 |  | Lactate dehydrogenase [Enzymatic activity/volume] in Serum or Plasma | 370.4 +/- 638.8 328.5 +/- 270.0 | 145 208 |  | 0.399 | 0.085 |  |
|  | 1 2 |  | Hemoglobin [Mass/volume] in Blood | 12.3 +/- 2.3 12.5 +/- 2.1 | 273 359 |  | 0.475 | 0.057 |  |
|  | 1 2 |  | Leukocytes [#/volume] in Blood | 27.3 +/- 273.8 24.7 +/- 258.8 | 246 315 |  | 0.907 | 0.010 |  |
| **Cohort 1 (N = 449) and cohort 2 (N = 449) characteristics after propensity score matching** | | | | | | | | | |
| **Demographics** | | | | | | | | |  |
|  | Cohort | |  | Mean ± SD | Patients |  | P-Value | Std diff. |  |
|  | 1 2 |  | Age at Index | 47.2 +/- 11.0 68.7 +/- 6.0 | 449 449 |  | <0.001 | 2.418 |  |
|  | 1 2 |  | White |  | 288 291 |  | 0.834 | 0.014 |  |
|  | 1 2 |  | Unknown Race |  | 64 63 |  | 0.924 | 0.006 |  |
|  | 1 2 |  | Female |  | 197 195 |  | 0.893 | 0.009 |  |
|  | 1 2 |  | Black or African American |  | 79 81 |  | 0.862 | 0.012 |  |
|  | 1 2 |  | Male |  | 252 254 |  | 0.893 | 0.009 |  |
|  | 1 2 |  | Asian |  | 10 10 |  | 1 | <0.001 |  |
| **Laboratory** | | | | | | | | |  |
|  | Cohort | |  | Mean ± SD | Patients |  | P-Value | Std diff. |  |
|  | 1 2 |  | Lactate dehydrogenase [Enzymatic activity/volume] in Serum or Plasma | 381.8 +/- 670.5 341.7 +/- 304.9 | 131 136 |  | 0.528 | 0.077 |  |
|  | 1 2 |  | Hemoglobin [Mass/volume] in Blood | 12.4 +/- 2.3 12.3 +/- 2.1 | 240 237 |  | 0.602 | 0.048 |  |
|  | 1 2 |  | Leukocytes [#/volume] in Blood | 30.2 +/- 294.2 32.8 +/- 319.2 | 213 207 |  | 0.931 | 0.008 |  |

**B) LDH**

| **Cohort 1 (N = 253) and cohort 2 (N = 222) characteristics before propensity score matching** | | | | | | | | | |
| --- | --- | --- | --- | --- | --- | --- | --- | --- | --- |
|  | **Demographics** | | | | | | | | |
|  |  | Cohort | |  | Mean ± SD | Patients |  | P-Value | Std diff. |
|  |  | 1 2 |  | Age at Index | 58.1 +/- 13.6 60.3 +/- 13.5 | 216 211 |  | 0.096 | 0.161 |
|  |  | 1 2 |  | White |  | 164 126 |  | <0.001 | 0.352 |
|  |  | 1 2 |  | Unknown Race |  | 13 28 |  | 0.011 | 0.248 |
|  |  | 1 2 |  | Female |  | 81 92 |  | 0.199 | 0.125 |
|  |  | 1 2 |  | Black or African American |  | 32 49 |  | 0.027 | 0.215 |
|  |  | 1 2 |  | Male |  | 135 119 |  | 0.199 | 0.125 |
|  |  | 1 2 |  | Asian |  | 10 10 |  | 0.957 | 0.005 |
|  | **Laboratory** | | | | | | | | |
|  |  | Cohort | |  | Mean ± SD | Patients |  | P-Value | Std diff. |
|  |  | 1 2 |  | Lactate dehydrogenase [Enzymatic activity/volume] in Serum or Plasma | 193.7 +/- 45.8 496.3 +/- 561.4 | 216 211 |  | <0.001 | 0.760 |
|  |  | 1 2 |  | Hemoglobin [Mass/volume] in Blood | 12.4 +/- 2.3 11.6 +/- 2.2 | 211 199 |  | 0.001 | 0.336 |
|  |  | 1 2 |  | Leukocytes [#/volume] in Blood | 54.6 +/- 451.3 14.3 +/- 17.0 | 193 176 |  | 0.238 | 0.126 |
| **Cohort 1 (N = 156) and cohort 2 (N = 156) characteristics after propensity score matching** | | | | | | | | | |
|  | **Demographics** | | | | | | | | |
|  |  | Cohort | |  | Mean ± SD | Patients |  | P-Value | Std diff. |
|  |  | 1 2 |  | Age at Index | 60.2 +/- 12.7 60.3 +/- 12.9 | 156 156 |  | 0.954 | 0.007 |
|  |  | 1 2 |  | White |  | 111 113 |  | 0.801 | 0.028 |
|  |  | 1 2 |  | Unknown Race |  | 13 12 |  | 0.835 | 0.024 |
|  |  | 1 2 |  | Female |  | 67 67 |  | 1 | <0.001 |
|  |  | 1 2 |  | Black or African American |  | 27 26 |  | 0.880 | 0.017 |
|  |  | 1 2 |  | Male |  | 89 89 |  | 1 | <0.001 |
|  |  | 1 2 |  | Asian |  | 10 10 |  | 1 | <0.001 |
|  | **Laboratory** | | | | | | | | |
|  |  | Cohort | |  | Mean ± SD | Patients |  | P-Value | Std diff. |
|  |  | 1 2 |  | Lactate dehydrogenase [Enzymatic activity/volume] in Serum or Plasma | 198.2 +/- 47.5 502.4 +/- 616.6 | 156 156 |  | <0.001 | 0.696 |
|  |  | 1 2 |  | Hemoglobin [Mass/volume] in Blood | 12.3 +/- 2.2 11.7 +/- 2.3 | 152 152 |  | 0.028 | 0.253 |
|  |  | 1 2 |  | Leukocytes [#/volume] in Blood | 73.6 +/- 535.0 14.4 +/- 17.6 | 137 139 |  | 0.194 | 0.156 |

**C) Anemia (female)**

| **Cohort 1 (N = 194) and cohort 2 (N = 155) characteristics before propensity score matching** | | | | | | | | | |
| --- | --- | --- | --- | --- | --- | --- | --- | --- | --- |
|  | **Demographics** | | | | | | | | |
|  |  | Cohort | |  | Mean ± SD | Patients |  | P-Value | Std diff. |
|  |  | 1 2 |  | Age at Index | 60.5 +/- 13.1 58.4 +/- 14.6 | 175 146 |  | 0.196 | 0.145 |
|  |  | 1 2 |  | White |  | 118 81 |  | 0.028 | 0.247 |
|  |  | 1 2 |  | Unknown Race |  | 15 13 |  | 0.916 | 0.012 |
|  |  | 1 2 |  | Female |  | 175 146 |  | -- | -- |
|  |  | 1 2 |  | Black or African American |  | 35 41 |  | 0.090 | 0.190 |
|  |  | 1 2 |  | Male |  | 0 0 |  | -- | -- |
|  |  | 1 2 |  | Asian |  | 10 10 |  | 0.675 | 0.047 |
|  | **Laboratory** | | | | | | | | |
|  |  | Cohort | |  | Mean ± SD | Patients |  | P-Value | Std diff. |
|  |  | 1 2 |  | Lactate dehydrogenase [Enzymatic activity/volume] in Serum or Plasma | 284.9 +/- 159.0 430.7 +/- 586.4 | 88 76 |  | 0.026 | 0.339 |
|  |  | 1 2 |  | Hemoglobin [Mass/volume] in Blood | 13.2 +/- 1.1 10.2 +/- 1.4 | 175 146 |  | <0.001 | 2.468 |
|  |  | 1 2 |  | Leukocytes [#/volume] in Blood | 37.7 +/- 342.5 49.5 +/- 414.0 | 157 123 |  | 0.795 | 0.031 |
| **Cohort 1 (N = 122) and cohort 2 (N = 122) characteristics after propensity score matching** | | | | | | | | | |
|  | **Demographics** | | | | | | | | |
|  |  | Cohort | |  | Mean ± SD | Patients |  | P-Value | Std diff. |
|  |  | 1 2 |  | Age at Index | 60.3 +/- 13.8 59.7 +/- 14.0 | 122 122 |  | 0.709 | 0.048 |
|  |  | 1 2 |  | White |  | 76 74 |  | 0.792 | 0.034 |
|  |  | 1 2 |  | Unknown Race |  | 10 11 |  | 0.819 | 0.029 |
|  |  | 1 2 |  | Female |  | 122 122 |  | -- | -- |
|  |  | 1 2 |  | Black or African American |  | 30 29 |  | 0.881 | 0.019 |
|  |  | 1 2 |  | Male |  | 0 0 |  | -- | -- |
|  |  | 1 2 |  | Asian |  | 10 10 |  | 1 | <0.001 |
|  | **Laboratory** | | | | | | | | |
|  |  | Cohort | |  | Mean ± SD | Patients |  | P-Value | Std diff. |
|  |  | 1 2 |  | Lactate dehydrogenase [Enzymatic activity/volume] in Serum or Plasma | 279.7 +/- 165.2 365.0 +/- 193.8 | 59 63 |  | 0.010 | 0.474 |
|  |  | 1 2 |  | Hemoglobin [Mass/volume] in Blood | 13.1 +/- 1.0 10.2 +/- 1.3 | 122 122 |  | <0.001 | 2.429 |
|  |  | 1 2 |  | Leukocytes [#/volume] in Blood | 51.6 +/- 416.8 12.5 +/- 18.2 | 106 111 |  | 0.325 | 0.132 |

**D) Anemia (male)**

| **Cohort 1 (N = 228) and cohort 2 (N = 193) characteristics before propensity score matching** | | | | | | | | | |
| --- | --- | --- | --- | --- | --- | --- | --- | --- | --- |
|  | **Demographics** | | | | | | | | |
|  |  | Cohort | |  | Mean ± SD | Patients |  | P-Value | Std diff. |
|  |  | 1 2 |  | Age at Index | 58.7 +/- 13.2 59 +/- 14.9 | 210 176 |  | 0.847 | 0.020 |
|  |  | 1 2 |  | White |  | 164 115 |  | 0.005 | 0.286 |
|  |  | 1 2 |  | Unknown Race |  | 18 13 |  | 0.670 | 0.044 |
|  |  | 1 2 |  | Female |  | 0 0 |  | -- | -- |
|  |  | 1 2 |  | Black or African American |  | 23 44 |  | <0.001 | 0.372 |
|  |  | 1 2 |  | Male |  | 210 176 |  | -- | -- |
|  |  | 1 2 |  | Asian |  | 10 10 |  | 0.685 | 0.041 |
|  | **Laboratory** | | | | | | | | |
|  |  | Cohort | |  | Mean ± SD | Patients |  | P-Value | Std diff. |
|  |  | 1 2 |  | Lactate dehydrogenase [Enzymatic activity/volume] in Serum or Plasma | 282.6 +/- 213.2 419.3 +/- 650.3 | 111 101 |  | 0.037 | 0.282 |
|  |  | 1 2 |  | Hemoglobin [Mass/volume] in Blood | 14.4 +/- 1.2 10.9 +/- 1.6 | 210 176 |  | <0.001 | 2.464 |
|  |  | 1 2 |  | Leukocytes [#/volume] in Blood | 9.4 +/- 6.7 11.2 +/- 12.7 | 180 145 |  | 0.095 | 0.181 |
| **Cohort 1 (N = 150) and cohort 2 (N = 150) characteristics after propensity score matching** | | | | | | | | | |
|  | **Demographics** | | | | | | | | |
|  |  | Cohort | |  | Mean ± SD | Patients |  | P-Value | Std diff. |
|  |  | 1 2 |  | Age at Index | 58.6 +/- 13.5 60.1 +/- 14.9 | 150 150 |  | 0.388 | 0.100 |
|  |  | 1 2 |  | White |  | 112 112 |  | 1 | <0.001 |
|  |  | 1 2 |  | Unknown Race |  | 12 13 |  | 0.835 | 0.024 |
|  |  | 1 2 |  | Female |  | 0 0 |  | -- | -- |
|  |  | 1 2 |  | Black or African American |  | 22 21 |  | 0.869 | 0.019 |
|  |  | 1 2 |  | Male |  | 150 150 |  | -- | -- |
|  |  | 1 2 |  | Asian |  | 10 10 |  | 1 | <0.001 |
|  | **Laboratory** | | | | | | | | |
|  |  | Cohort | |  | Mean ± SD | Patients |  | P-Value | Std diff. |
|  |  | 1 2 |  | Lactate dehydrogenase [Enzymatic activity/volume] in Serum or Plasma | 291.9 +/- 228.1 442.5 +/- 700.3 | 91 86 |  | 0.053 | 0.289 |
|  |  | 1 2 |  | Hemoglobin [Mass/volume] in Blood | 14.3 +/- 1.1 11.0 +/- 1.6 | 150 150 |  | <0.001 | 2.410 |
|  |  | 1 2 |  | Leukocytes [#/volume] in Blood | 9.4 +/- 7.4 11.7 +/- 13.5 | 126 125 |  | 0.097 | 0.210 |

**E) White blood count**

| **Cohort 1 (N = 500) and cohort 2 (N = 202) characteristics before propensity score matching** | | | | | | | | | |
| --- | --- | --- | --- | --- | --- | --- | --- | --- | --- |
|  | **Demographics** | | | | | | | | |
|  |  | Cohort | |  | Mean ± SD | Patients |  | P-Value | Std diff. |
|  |  | 1 2 |  | Age at Index | 58.2 +/- 14.7 60.1 +/- 13.1 | 455 184 |  | 0.120 | 0.139 |
|  |  | 1 2 |  | White |  | 318 120 |  | 0.249 | 0.100 |
|  |  | 1 2 |  | Unknown Race |  | 35 14 |  | 0.971 | 0.003 |
|  |  | 1 2 |  | Female |  | 200 80 |  | 0.912 | 0.010 |
|  |  | 1 2 |  | Black or African American |  | 84 39 |  | 0.427 | 0.069 |
|  |  | 1 2 |  | Male |  | 255 104 |  | 0.912 | 0.010 |
|  |  | 1 2 |  | Asian |  | 11 10 |  | 0.053 | 0.156 |
|  | **Laboratory** | | | | | | | | |
|  |  | Cohort | |  | Mean ± SD | Patients |  | P-Value | Std diff. |
|  |  | 1 2 |  | Lactate dehydrogenase [Enzymatic activity/volume] in Serum or Plasma | 300.5 +/- 378.3 445.2 +/- 606.2 | 240 107 |  | 0.007 | 0.286 |
|  |  | 1 2 |  | Hemoglobin [Mass/volume] in Blood | 12.4 +/- 2.2 12.1 +/- 2.2 | 447 180 |  | 0.070 | 0.160 |
|  |  | 1 2 |  | Leukocytes [#/volume] in Blood | 6.7 +/- 2.8 68.1 +/- 461.1 | 455 184 |  | 0.005 | 0.189 |
| **Cohort 1 (N = 181) and cohort 2 (N = 181) characteristics after propensity score matching** | | | | | | | | | |
|  | **Demographics** | | | | | | | | |
|  |  | Cohort | |  | Mean ± SD | Patients |  | P-Value | Std diff. |
|  |  | 1 2 |  | Age at Index | 60.9 +/- 13.5 60.0 +/- 13.2 | 181 181 |  | 0.518 | 0.068 |
|  |  | 1 2 |  | White |  | 132 120 |  | 0.170 | 0.145 |
|  |  | 1 2 |  | Unknown Race |  | 13 14 |  | 0.841 | 0.021 |
|  |  | 1 2 |  | Female |  | 77 77 |  | 1 | <0.001 |
|  |  | 1 2 |  | Black or African American |  | 25 39 |  | 0.054 | 0.204 |
|  |  | 1 2 |  | Male |  | 104 104 |  | 1 | <0.001 |
|  |  | 1 2 |  | Asian |  | 10 10 |  | 1 | <0.001 |
|  | **Laboratory** | | | | | | | | |
|  |  | Cohort | |  | Mean ± SD | Patients |  | P-Value | Std diff. |
|  |  | 1 2 |  | Lactate dehydrogenase [Enzymatic activity/volume] in Serum or Plasma | 335.4 +/- 530.0 445.8 +/- 611.7 | 110 105 |  | 0.158 | 0.193 |
|  |  | 1 2 |  | Hemoglobin [Mass/volume] in Blood | 12.1 +/- 2.3 12.1 +/- 2.2 | 178 177 |  | 0.874 | 0.017 |
|  |  | 1 2 |  | Leukocytes [#/volume] in Blood | 6.9 +/- 3.0 68.9 +/- 464.9 | 181 181 |  | 0.074 | 0.189 |

**F) Race**

| **Cohort 1 (N = 803) and cohort 2 (N = 219) characteristics before propensity score matching** | | | | | | | | | |
| --- | --- | --- | --- | --- | --- | --- | --- | --- | --- |
|  | **Demographics** | | | | | | | | |
|  |  | Cohort | |  | Mean ± SD | Patients |  | P-Value | Std diff. |
|  |  | 1 2 |  | Age at Index | 61.2 +/- 13.3 53.7 +/- 14.0 | 725 210 |  | <0.001 | 0.547 |
|  |  | 1 2 |  | White |  | 725 0 |  | <0.001 | -- |
|  |  | 1 2 |  | Unknown Race |  | 0 0 |  | -- | -- |
|  |  | 1 2 |  | Female |  | 304 113 |  | 0.002 | 0.239 |
|  |  | 1 2 |  | Black or African American |  | 0 210 |  | <0.001 | -- |
|  |  | 1 2 |  | Male |  | 421 97 |  | 0.002 | 0.239 |
|  |  | 1 2 |  | Asian |  | 0 0 |  | -- | -- |
|  | **Laboratory** | | | | | | | | |
|  |  | Cohort | |  | Mean ± SD | Patients |  | P-Value | Std diff. |
|  |  | 1 2 |  | Lactate dehydrogenase [Enzymatic activity/volume] in Serum or Plasma | 345.9 +/- 538.9 346.5 +/- 162.1 | 236 56 |  | 0.993 | 0.002 |
|  |  | 1 2 |  | Hemoglobin [Mass/volume] in Blood | 12.7 +/- 2.2 11.7 +/- 2.0 | 410 115 |  | <0.001 | 0.481 |
|  |  | 1 2 |  | Leukocytes [#/volume] in Blood | 9.9 +/- 10.8 10.5 +/- 10.0 | 366 101 |  | 0.635 | 0.054 |
| **Cohort 1 (N = 202) and cohort 2 (N = 202) characteristics after propensity score matching** | | | | | | | | | |
|  | **Demographics** | | | | | | | | |
|  |  | Cohort | |  | Mean ± SD | Patients |  | P-Value | Std diff. |
|  |  | 1 2 |  | Age at Index | 54.5 +/- 13.8 54.9 +/- 12.8 | 202 202 |  | 0.720 | 0.036 |
|  |  | 1 2 |  | White |  | 202 0 |  | <0.001 | -- |
|  |  | 1 2 |  | Unknown Race |  | 0 0 |  | -- | -- |
|  |  | 1 2 |  | Female |  | 104 107 |  | 0.765 | 0.030 |
|  |  | 1 2 |  | Black or African American |  | 0 202 |  | <0.001 | -- |
|  |  | 1 2 |  | Male |  | 98 95 |  | 0.765 | 0.030 |
|  |  | 1 2 |  | Asian |  | 0 0 |  | -- | -- |
|  | **Laboratory** | | | | | | | | |
|  |  | Cohort | |  | Mean ± SD | Patients |  | P-Value | Std diff. |
|  |  | 1 2 |  | Lactate dehydrogenase [Enzymatic activity/volume] in Serum or Plasma | 450.9 +/- 951.1 346.5 +/- 162.1 | 63 56 |  | 0.419 | 0.153 |
|  |  | 1 2 |  | Hemoglobin [Mass/volume] in Blood | 12.7 +/- 2.3 11.7 +/- 2.0 | 113 111 |  | <0.001 | 0.506 |
|  |  | 1 2 |  | Leukocytes [#/volume] in Blood | 10.5 +/- 15.6 10.5 +/- 10.1 | 102 98 |  | 0.968 | 0.006 |

**G) Sex**

| **Cohort 1 (N = 568) and cohort 2 (N = 709) characteristics before propensity score matching** | | | | | | | | | |
| --- | --- | --- | --- | --- | --- | --- | --- | --- | --- |
|  | **Demographics** | | | | | | | | |
|  |  | Cohort | |  | Mean ± SD | Patients |  | P-Value | Std diff. |
|  |  | 1 2 |  | Age at Index | 59.3 +/- 13.9 59.1 +/- 13.8 | 499 612 |  | 0.844 | 0.012 |
|  |  | 1 2 |  | White |  | 304 421 |  | 0.006 | 0.165 |
|  |  | 1 2 |  | Unknown Race |  | 58 76 |  | 0.686 | 0.024 |
|  |  | 1 2 |  | Female |  | 499 0 |  | <0.001 | -- |
|  |  | 1 2 |  | Black or African American |  | 113 97 |  | 0.004 | 0.173 |
|  |  | 1 2 |  | Male |  | 0 612 |  | <0.001 | -- |
|  |  | 1 2 |  | Asian |  | 12 10 |  | 0.359 | 0.055 |
|  | **Laboratory** | | | | | | | | |
|  |  | Cohort | |  | Mean ± SD | Patients |  | P-Value | Std diff. |
|  |  | 1 2 |  | Lactate dehydrogenase [Enzymatic activity/volume] in Serum or Plasma | 353.7 +/- 409.0 332.5 +/- 424.7 | 180 246 |  | 0.604 | 0.051 |
|  |  | 1 2 |  | Hemoglobin [Mass/volume] in Blood | 12.0 +/- 1.9 12.9 +/- 2.3 | 313 389 |  | <0.001 | 0.403 |
|  |  | 1 2 |  | Leukocytes [#/volume] in Blood | 42.0 +/- 377.0 10.2 +/- 9.7 | 277 338 |  | 0.122 | 0.119 |
| **Cohort 1 (N = 467) and cohort 2 (N = 467) characteristics after propensity score matching** | | | | | | | | | |
|  | **Demographics** | | | | | | | | |
|  |  | Cohort | |  | Mean ± SD | Patients |  | P-Value | Std diff. |
|  |  | 1 2 |  | Age at Index | 59.5 +/- 13.8 59.6 +/- 13.3 | 467 467 |  | 0.851 | 0.012 |
|  |  | 1 2 |  | White |  | 303 313 |  | 0.490 | 0.045 |
|  |  | 1 2 |  | Unknown Race |  | 58 49 |  | 0.355 | 0.061 |
|  |  | 1 2 |  | Female |  | 467 0 |  | <0.001 | -- |
|  |  | 1 2 |  | Black or African American |  | 92 90 |  | 0.869 | 0.011 |
|  |  | 1 2 |  | Male |  | 0 467 |  | <0.001 | -- |
|  |  | 1 2 |  | Asian |  | 10 10 |  | 1 | <0.001 |
|  | **Laboratory** | | | | | | | | |
|  |  | Cohort | |  | Mean ± SD | Patients |  | P-Value | Std diff. |
|  |  | 1 2 |  | Lactate dehydrogenase [Enzymatic activity/volume] in Serum or Plasma | 355.4 +/- 410.9 320.7 +/- 436.7 | 178 183 |  | 0.437 | 0.082 |
|  |  | 1 2 |  | Hemoglobin [Mass/volume] in Blood | 12.1 +/- 1.9 12.9 +/- 2.3 | 290 303 |  | <0.001 | 0.377 |
|  |  | 1 2 |  | Leukocytes [#/volume] in Blood | 45.2 +/- 393.6 10.6 +/- 10.5 | 254 269 |  | 0.150 | 0.124 |
